# Function of cGMP-dependent protein kinase II in volume expansion-induced diuresis

**DOI:** 10.1186/2050-6511-14-S1-P63

**Published:** 2013-08-29

**Authors:** Andrea Schramm, Elisabeth Schinner, Jens Schlossmann

**Affiliations:** 1 Department of Pharmacology and Toxicology,University of Regensburg, Germany

## Background

The field of cGMP/cGKII-mediated mechanisms regarding kidney function is rather unexplored. ANP, as the major upstream regulator of cGKII, is known to cause diuresis and natriuresis by renal vascular as well as direct tubular effects. The collecting duct (CD) is thought to be the main target site of ANP in the kidney; an inhibition of Na^+^- reabsorption was shown for outer and inner medullary CD [1-3]. In contrast, the effect of ANP on fluid reabsorption in CD is discussed very controversially. Short-term regulation of Aquaporin 2 (AQP2), the predominant apical water channel in the CD, occurs mainly via membrane insertion/excision. Stimulation as well as inhibition of AQP2-trafficking upon ANP-administration have been reported [4,5]. However, the downstream effectors of ANP regarding fluid- and ion-absorption have not been elucidated so far.

## Results

We investigated if renal parameters in different conditions (basal, salt diets, water load) are dependent on cGKII by using a genetically modified mouse model (cGKII-KO). We could not detect any differences between WT and cGKII-KO during basal conditions, except for a slightly decreased urine output and a significantly lowered amount of creatinine in urine during a normal salt diet.

When mice are subjected to a volume expansion (performed by application of a 10mM glucose-solution (3% of BW) via feeding needle), WT mice exhibit a potent diuresis. In contrast, urine volume is decreased significantly in cGKII-KO. Furthermore, Na^+^-excretion is also significantly lowered in cGKII-KO. This effect can be abolished by similar administration of Amiloride.

## Conclusion

During different salt loads, cGKII is proposed to be involved only to a minor extent in regulating the renal concentration ability. In contrast, cGKII-KO mice are not able to handle an acute volume load. Our results suggest that membrane insertion of AQP2 is inhibited by cGMP/cGKII. Furthermore, we could eludicate that cGKII is an important regulator of sodium reabsorption by ENaC.
